# Childhood Trauma, Personality, and Substance Use Disorder: The Development of a Neuropsychoanalytic Addiction Model

**DOI:** 10.3389/fpsyt.2020.00531

**Published:** 2020-06-09

**Authors:** Jürgen Fuchshuber, Human Friedrich Unterrainer

**Affiliations:** ^1^Center for Integrative Addiction Research (CIAR), Grüner Kreis Society, Vienna, Austria; ^2^University Clinic for Psychiatry and Psychotherapeutic Medicine, Medical University Graz, Graz, Austria; ^3^Department of Religious Studies, University of Vienna, Vienna, Austria

**Keywords:** substance use disorder, psychoanalysis, neuropsychoanalysis, childhood trauma, affect regulation

## Abstract

**Background:**

While traditional psychoanalysis has been criticized as insufficient for the treatment of substance use disorder (SUD), recent progress in the field of neuropsychoanalysis has generated new and promising hypotheses regarding its etiology. However, empirical research applying this framework has been sparse.

**Aim and Scope:**

The present overview aims at developing and empirically validating a neuroscientifically informed psychodynamic framework regarding the etiology of SUD. For this purpose, this review provides a concise overview of the most relevant historical and contemporary psychoanalytic theories on SUD etiology. Furthermore, the original research summarized in this paper consists of three studies investigating connections between childhood trauma, primary emotions, personality structure and attachment, as well as their relation to SUD development and treatment.

**Conclusions:**

The results highlight the empirical validity of the neuropsychoanalytic approach towards SUD etiology. In particular, the findings underscore the conceptualization of SUD as a disorder related to dysfunctional attachment and affect regulation abilities especially linked to increased SADNESS and ANGER dispositions, which mediated the relationship between SUD and traumatic childhood relationships. Based on these findings, a refined model of SUD etiology is proposed, which should be tested in future studies.

## Introduction

Substance use disorders (SUD), commonly referred to as addictions, are commonly defined as continuing, pathological, and compelling urges to consume one or more psychoactive substances, despite detrimental effects for oneself and others (1). Despite a “war on drugs”, which has raged for almost 50 years with the aim of achieving a drug-free society, SUDs are still one of the most common psychiatric disorders worldwide. As stated in the World Drug Report 2017, problematic substance use and SUD presently affects about 29.5 million people (2). The consequences of this disorder not only impact individual health, but also significantly strain the public health systems and inflict a toll upon society not often reported upon.

Considering the wide spectrum of psychoanalytic theories concerned with SUD etiology following Bilitza (1) and Rost (3), it is feasible to structure these approaches into the categories *drive theory*, *ego psychology*, and *object relations theory*. Furthermore, etiological models related to the work of Jacque Lacan, which have been traditionally neglected in reviews concerned with this topic, might be summarized under the umbrella term *structural psychoanalysis*. While all of these paradigms are associated content-wise, they often differ substantially in their emphases of specific etiological aspects.

The following will give a brief overview of the most important theoretical concepts of these schools of thought. Furthermore, this paper will review several contemporary SUD approaches of attachment theory and neuropsychoanalysis, which can be seen as further developments of classic psychoanalysis. Finally, we intend to give an overview of recent empirical research undertaken by our research group, which aimed to develop and validate an integrative neuropsychodynamic approach to SUD etiology.

### Classical Drive Theory Approaches to SUD Etiology

Despite his publication on cocaine (4), Freud did not develop a comprehensive theory regarding the development of addiction disorders. This fact might not only be explained by Freud’s serious nicotine addiction, leading to a lack of inner distance towards this topic, but also by his recommendation of cocaine as a cure for his morphine addicted friend Fleischl-Marxow, which significantly contributed to his friend’s early death in 1891 (5). Therefore, Freud’s notes on addiction are sparse and dispersed over several works. In his earliest remarks regarding addiction, Freud argues that chronic substance use might act as a substitute for sexual impulses and replace the “Ursucht” of infantile masturbation (6).

Later on, in his *Three essays on the theory of sexuality* Freud (7) highlights the importance of oral fixation regarding the etiology of addiction. Freud [(8), p.182] concludes: “It is not every child who sucks in this way. It may be assumed that those children do so in whom there is a constitutional intensification of the erotogenic significance of the labial region. If that significance persists, these same children when they are grown up will become epicures in kissing, will be inclined to perverse kissing, or, if males, will have a powerful motive for drinking and smoking.” The idea that oral fixation and a consecutive disposition towards oral regression plays a significant role in the etiology of SUDs is reiterated by a great proportion of later psychoanalytic theorists, including Clark (9), Abraham (10), Rado (11), Knight (12), Fenichel (13), Rosenfeld (14), Limentani (15), Khantzian (16), and Wurmser (17). These authors agree on the predominance of a regressive oral defense mode in response to conflicts, which emerges due to an interplay of unresolved traumatic childhood experiences and constitutional factors. The ego of addicts is seen as insufficiently developed and therefore unable to withstand the anxiety. Hence, the relationship between the patient and the psychoactive substance is interpreted as a representation of the fulfillment of primitive and ambivalent oral demands and phantasies (18).

Finally, linked to his controversial introduction of the death drive—originally proposed by Sabina Spielrein (19)—Freud (20) shifts the emphasis regarding addiction development from direct masturbatory satisfaction of the pleasure principle to the avoidance of unpleasure. This establishes the function of drugs as artificial defense mechanisms, helping the individual to cope with external and internal stressors and establishes a psychological state comparable with mania, that temporally restores the phase of infantile narcissism. This approach is further elaborated by Simmel (21, 22), who conceptualizes addiction as a chemically evoked manic defense against melancholy, as alcohol attacks and ultimately disconnects the destructive super-ego of the addict.

The essay “The psychological relation between sexuality and alcoholism” (10), which was originally published in 1908, was the first psychoanalytic contribution exclusively discussing alcoholism. As claimed by Abraham, alcohol addiction is not only associated with oral fixation but also initially aims to increase sexual performance. Hence, he understands alcohol as a symbol of male fertility and the semen of the father. However, he observed a gradual decrease of virility in the later stages of addiction which is accompanied by increased castration anxieties, leading to a gradual replacement of the genital sexual aim by the alcoholic beverage. In this context, alcohol is conceptualized as a fetish-linking addiction to perversion (see also 23 for further discussion). Moreover, he proposes that chronic alcohol consumption dissolves mature defense mechanisms like inhibition, repression, and sublimation, which facilitates the release of unconscious homosexuality and sadistic partial drives, a trope which is repeatedly reiterated in the early psychoanalytic literature dealing with SUDs (18).

The contributions of the Hungarian analyst Rado (11, 23), might be the most influential drive-theoretical considerations concerned with addiction etiology. His first essay (11) focused predominantly on the significance of oral fixation, which he linked to the term “alimentary orgasm”. This refers to orgasmic pleasure which is derived from the consumption and digestion of food. However, for the most part this concept gained little recognition from later theorists (24). Nevertheless, his second essay (23) is regarded as a seminal work for the later development of the ego-psychological conceptualization of this disorder (3). Rado’s starting point is his observation of two kinds of effects linked to drug consumption: A sedative and analgesic effect, reducing reluctance and pain, and a euphoria inducing and stimulating effect, generating pleasure. The latter is seen as more problematic regarding addiction development, as it is prone to gradually compete with sexual desire, especially in the case of restricted opportunities for sexual satisfaction (11). What is more, he claims that his addicted patients are characterized by a specific predisposition towards an “initial depression” or “tense depression” in response to frustrations (23). He defines this as the co-occurrence of both decreased tolerance of pain and extraordinary painful tensions. Therefore, drugs intervene in the insufficient affect regulation acting as an artificial stimuli barrier against both outwards and inwards frustrations. Referring to Freud’s (25) theory of infantile narcissism he claims that “[i]n the pharmacogenic elation the ego regains its original narcissistic stature” [(23); p.57]. For the subject, this appears even more compelling as it can be achieved without effort and without the help of the other. However, as the effects of the drugs wear off the blissful state is replaced by an aggravated version of the initial depression, enhanced by a sharpened contrast between real-ego and ideal-ego, feelings of guilt and an exacerbated fear of reality. This intensifies cravings for the next drug hit, setting in motion a manic-depressive vacuous cycle and gradually shifting the ego organization from a realistic to a pharmacological regime. In line with Abraham (10), he observes decreasing genital drives as succumbing to pharmacological desires, which revive castration anxieties and hence are gradually displaced by fears of the psychoactive substance dissipating and increasing homosexual desires. Finally, a defusion of drives succeed in releasing aggressive impulses, which result in increased feelings of guilt and a secondary need for punishment (23).

### Jouissance and Substance Use Disorder: The Lacanian Understanding of Addiction

While Lacan himself did not write much on addiction (26), in recent years scholars of Lacan contributed three major works on this subject [see: (27, 28)]. Following Freud’s (4) writings on cocaine, Loose (26) highlights the significance of vulnerability within the subject in SUD development as opposed to the pharmacological effects of the drug itself. Despite not denying the addictive properties of specific psychoactive substances, he argues that the SUD patient receives a subject-unique effect from the substance, which people who are not addicted do not experience. In this context, the concept of *jouissance* is given a central place in the Lacanian etiology of SUD (26, 28).

Initially, Lacan used the term *jouissance—*usually translated as enjoyment of judicial rights, property, and sexual orgasm—to designate feelings of enjoyment tied to the satisfaction of biological needs like hunger and sexuality (29). Subsequently, referring to Kojeve (30), he developed a juxtaposition which contrasted jouissance and pleasure. In this context, Lacan argues that the pleasure principle acts as a restriction of the subjects enjoyment (31). Simultaneously, the subject attempts to transgress this limitation aiming to go beyond the pleasure principle. However, the transgression of the pleasure principle leads to pain and death (32). The prohibition of *jouissance* according to the pleasure principle is inherent to the symbolic order introduced by the “Law/Name-of-the-Father” (French translation: nom du père) at the end of the oedipal situation (31). This implies the forgoing of identification as the imaginary phallus for the mother due to symbolic castration. Loose (26) argues that many addicts suspend the structural obstruction of *jouissance via* the administration of psychoactive substances, aiming at a *jouissance* which is “non-phallic”, non-linguistic, and independent of social attachments.

In correspondence to this, it is important to note that the Lacanian-framework proposes three different clinical structures which determine the function of the psychoactive substance for the addicted subject. Thereby, Lacan (33) differentiates between the (1) neurotic, (2) perverse, and (3) psychotic clinical structure, which is distinguished by their predominant mode of defense against the symbolic castration. In correspondence to this, (1) a neurotic structure is linked to repression; (2) a perverse structure is linked to disavowal; and (3) a psychotic structure is linked to dismissal of the symbolic order (31, 34). For neurotic and perverse subjects, these mechanisms result in a fundamental discontent with the pleasure principle. However, while what lies beyond the pleasure principle is “too much”, the unavailability of that which is beyond generates a sense of “never enough” (28).

Based on this idea, SUD is understood as a technique to manage *jouissance*. For a structurally neurotic or perverse subject SUD is associated with the release of an surplus *jouissance*, which would otherwise be obstructed by the symbolic order or “symbolic castration” (26). Thus, drugs play the role of an “object-cause-of-*jouissance*”, helping the subject to bypass the desire of the Other as a precondition for enjoyment, as required by the symbolic order. In contrast, for a psychotic subject the chronic consumption of psychoactive substances aims at limiting the surplus *jouissance*, which he/she experiences as invading, threatening, and often in the form of anxiety or pain (26). Therefore, within a psychotic structure drugs substitute the function usually executed by the symbolic order of language and the desire of the Other. Hence, the administration of psychoactive substances acts as a floodgate mechanism (28).

### Ego Psychological Approaches Towards SUD

In contrast to drive theory, ego psychology prioritizes the ego and its functions regarding the etiology of psychopathological phenomena. The origins of this psychoanalytic school can be traced back to Anna Freud’s (35) systematic categorization of defense mechanisms. This approach was then further elaborated upon in the writings of Heinz Hartmann (36), Margaret Mahler (37), Edith Jacobson (38), and Roy Schafer (39). In general, these authors agree that psychopathological symptoms are a result of defects within personality structure, which arise due to disruptions—like trauma, deprivation, or innate defects—during the development of the ego (40). In correspondence to this, personality structure is described as a set of ego-functions ensuring the maintenance of relationships to others and inner equilibrium (41). Thereby, personality structure and affects are compared to a container and its contents (42).

With regard to SUD, this “container” is too porous, causing the addicted patient to consume drugs to self-medicate unbearable affective states, iterating Freud’s (20) and Rado’s (23) writings on SUD etiology. Accordingly, Krystal and Raskin (43), Heigl-Evers (44), and Wurmser (17) underscore the significance of the addicts’ inability to regulate overwhelming, unpleasurable, and often undifferentiated affects. This is often linked to a disposition towards depression and anxiety which is often somatized, unverbalized, and experienced as physical pain. For example, Wurmser (17) emphasizes the role of narcissistic crises and intense feelings of depression, anxiety, rage, shame, and loneliness in SUD development, which he associates with traumatic childhood experiences and consequential conflicts between a malfunctioning ego and an archaic and sadistic super-ego. This results in an alliance between id and ego against the super-ego and the external world. This is in sharp contrast to neurotic disorders, which are understood as a pact between ego, super-ego, and the external word against the id. Furthermore, he proposed several defense mechanisms characteristic for SUD. These comprise splitting and denial of the internal and external reality, externalization of inner conflicts, and identification with the aggressor (17). However, drug abuse might also result from too-rigid affect defenses as well (43, 45). In this case, the addicted subject might take drugs in order to temporarily experience feelings of oneness with loved objects, otherwise unachievable in a sober state due to strict defenses against aggression towards the object.

What is more, Khantzian and Mack (46) emphasize the role of self-care in the etiology of treatment of SUD. This concept is related to reality testing and discernment and functionally acts as a protection against self-destructive tendencies and external dangers. Maltreatment experienced in childhood is able to disturb the development of this function, as feelings of anxiety and pain associated with early childhood trauma are often managed by the child neglecting its own feelings and self-worth. In adulthood, this internalized coping strategy might then predispose them to risky behavior like drug abuse (47, 48).

### Object Relations Theory of SUD Etiology

Psychoanalytic object relations theory emphasizes the importance of early caregiver-child relationships and the early childhood environment, which contrasts with the often rather monadic approach of ego psychology. Moreover, while the classic oedipal paradigm of psychoanalysis focuses on the development from dyadic to triadic relationships, object relations theory is often more interested in the development from a primary symbiotic-like state between child and caregiver to more mature object representations (3). Major contributions to this school of thought were made by Melanie Klein and Roland Fairbairn. While Klein developed her theoretical framework around Freud’s late drive/structure model, highlighting the role of aggression and the death drive, Fairbairn and his followers—like Guntrip and Winnicott—shifted the psychoanalytic paradigm to a relational/structure model, spotlighting the subjects’ need for interpersonal relations rather than the desire for sexual gratification (49). In correspondence to this, Fairbairn’s work is closely linked to the so-called Middle Group of British Analysts and strongly influenced John Bowlby’s development of attachment theory (50).

Influenced by the Kleinian approach to object relations theory, early considerations regarding addiction were developed by Glover (51). He linked drugs to Klein’s (52) concept of part-objects, defined as something which is perceived as both single body parts (breast, penis, feces, etc.) and their symbolic equivalents (e.g. persecutory, soothing, benevolent) (53), existing on the border between the internal and external world. What is more, he stresses the etiological significance of specific phantasies fueled by primitive sadism, hate and aggression, as well as associated paranoid states. The central phantasy system of addiction disorders is characterized by Glover [(51); p.38] as: “[… ] a condensation of two primary systems, one in which the child attacks (later restores) organs in the mother´s body, and one in which the mother attacks (later restores) organs in the child´s body.” In this context, the influence of aggression in addiction etiology is proposed as less severe than in psychotic disorders, but more intense than in neurotic disorders. Furthermore, Glover argues that the addicted patients’ choice of drug is influenced by the intensity of destructive impulses, with more aggression linked to the selection of more hazardous substances. Therefore, in contrast to Abraham (10), Glover proposes that drugs of abuse symbolize not only (libidinously charged) semen but also (sadistically charged) feces. Consequently, the drug is perceived by the patient as an oscillating good and bad part-object. This is linked to an internalized and ambivalently loved mother (part-) object, which at its core is hated and felt as a hostile foreign body. Thus, the patient aims at destroying this bad and poisonous inner object by means of substance consumption, while the good representations of the self and others are projected onto the world, in an attempt to protect them against the individual’s own sadistic impulses. Correspondingly, drug addiction is functionally understood as a simultaneous defense against psychotic fragmentation linked to regression towards the paranoid-schizoid position, as described by Klein (52), and a destructive self-medication against painful melancholic intrapsychic conflict associated with a progression towards the depressive position. Hence, Glover understands drug addictions essentially as a transitional state between psychosis and neurosis, and a compromise between the projected aggression linked to paranoid states and introjected aggression linked to depressive states.

The Kleinian approach is supplemented by Rosenfeld (14), who claims that addiction development is centered around a defensive manic formation involving idealizing, identification with ideal objects and denial of persecutory fears and depressive anxiety. Moreover, drug intake is interpreted as an identification with sadistic and destructive objects, which persecute the good representations of objects and the self in order to achieve omnipotent control of them. This enables the patient to act out sadistic impulses without concerns, feelings of guilt, or control by the super-ego. However, for Rosenfeld drug intake also serves a depressive and masochistic function, as the substance symbolizes a dead or damaged object which the patient feels obligated to incorporate and ultimately identify with, due to intense feelings of guilt.

In summary, the Kleinian model of SUD seems to be more pessimistic than the concepts of ego-psychology. In the view of Kleinian object relation theory, the inner landscape of the SUD patient is scattered and archaic. While ego psychology emphasizes the self-medication aspects of SUD, object relations theory highlights self-destructive impulses as the central driving force in its etiology. In correspondence to this, both Glover (51) and Rosenfeld (14) describe a predominance of a rather primitive personality organization, fixated between the early paranoid-schizoid position and the later depressive position, and dominated by splitting and related mechanisms.

An attempt at approaching SUD etiology from an object relations theory perspective linked to the Middle Group of British Analysts was made by Krystal and Raskin (43) and Luerssen (54). These authors associated drug abuse with Winnicott’s (55) concept of the transitional object. Winnicott described transitional objects as a materialistic object, bearing a significant role for infants—usually between 4 and 12 months of age—particularly while falling asleep. These objects (e.g. a blanket or towel) are important in the development of the infant’s autonomy, as they substitute the calming qualities of the parents and, hence, are utilized in their absence. In turn, this permits a gradual transition between the early oral relationships with the caregiver and the later internalization of more mature object relations.

Accordingly, the drug abusing individual, who is unable to build stable inner object representations, employs the drug as a soothing and idealized object (43, 54). However, in contrast to the transitional object described by Winnicott, the psychoactive substance does not stay on the border between the external and internal world but oscillates. The addicted individual tries to incorporate the object in an attempt to overcome his severe fear of abundance. However, this introjection necessarily fails, due to the transformation of the drug into a persecutory object within the addict’s body. In correspondence to this, Rost (3) argues that the relationship between SUD patient and drug mirrors the unintegrated ambivalence of the early infant-caregiver relationship, split between separation anxiety and need for autonomy, as well as love and hate.

Vital impulses in the advancement of object relation SUD theory can be drawn from the work of Otto Kernberg. Strongly influenced by Melanie Klein, Edith Jacobson, and Roland Fairbairn, he integrated findings of Kleinian object psychology, ego-psychology, and the object relations theory of the Middle Group into a cohesive theoretical framework (49). Furthermore, he argues for the necessity of (quantitative) empirical and neuroscientific research in order to further the advance of psychoanalysis as a science (56, 57).

Kernberg (58, 59) developed a model of personality organization which differentiates between three theoretically and empirically connected levels of functioning: (1) Coherence of identity, denoting the cohesion of differentiated and integrated representations of oneself and others; (2) Maturity of defense mechanisms, meaning the ability to cope with internal and external conflicts in a functional way; (3) Ability to test reality, describing the competence to differentiate between internal and external stimuli. A constellation of a predominance of primitive defenses like splitting and related mechanisms, severe identity diffusion a relatively functional capacity to test reality, is summarized in the concept of “borderline organization”, which is closely related to Melanie Klein’s concept of the paranoid-schizoid position (56), and theoretically related but not identical with the diagnosis of borderline personality disorder (BPD) (60). In contrast, a decreased facility for reality testing, due to fused representations of the self and objects, is linked to a psychotic personality organization, while individuals with a neurotic personality organization show only marginal deficits in all three areas of functioning (61).

Regarding SUD and impulse neurosis (kleptomania, psychogenic obesity, etc.), Kernberg (60) observed chronic repetitions of instinct driven behaviors, which are experienced as ego dystonic in most life situations, but very gratifying and ego syntonic during episodes of acting out. In line with the SUD theory of Kleinian object psychology (14, 51), SUD etiology was linked to the splitting defense mechanism, which for Kernberg (58, 60), is the essential defense mechanism of the borderline personality organization. According to Kernberg (60), splitting refers to the active separation of internalizations with opposite—meaning libidinal or aggressive—affective quality. This process obstructs the integration or fusion of introjections and identifications with contrary properties and serves as the main source of neutralization of aggression. Furthermore, he argues in line with Jacobson (38, 62), that the process of neutralization, which is linked to the prefrontal and anterior cingulate cortex (56, 63), provides crucial energy for ego development. Hence, an impaired ego is predisposed to use splitting, as this mechanism requires less energy than more mature forms of defense like repression. In turn, this triggers in a vicious circle of reciprocal reinforcement between a fragile ego and excessive use of splitting. This intrapsychic process is linked to substantial deficits in affect integration and regulation and, therefore, manifests in symptoms associated with low impulse control, like addictive behaviors or self-harming behavior, which at the time of their expression are experienced as ego syntonic. Like repression, splitting is not an isolated mechanism but occurs simultaneously with other so called primitive defense mechanisms (60). These are comprised of: Primitive idealization, projective identification, denial and omnipotence and devaluation.

Furthermore, the persistence of splitting obstructs the development of a coherent identity marked by an integrated (“good” and “bad”) sense of self and others. This fosters the syndrome of identity diffusion, which is characterized by dissociated representations of the self and others into multiple segments of idealized and persecutory representations (64).

In correspondence to this, SUD can be seen as a coping strategy against painful and chaotic affective states, resulting from a borderline personality organization. However, this strategy creates a vicious circle, as the use of psychoactive substances further fosters the regression of ego functions and the fragmentation of its underlying object relations.

Recent quantitative-empirical studies, which investigated the relationship between SUD and personality organization, support the assumed association with a borderline personality organization (65, 66). Hiebler-Ragger et al. (65), applied the Borderline Personality Inventory [BPI; (67)] and compared healthy controls with inpatients diagnosed with SUD. These findings also suggest that SUD patients showed medium to large differences in almost every personality structure dimension measured with the BPI, including increased “identity diffusion”, “primitive defenses”, “fear of fusion”, and overall “structural deficits”. In contrast, the dimension “reality testing”, indicating a psychotic perception of reality, revealed only small differences between healthy and inpatient participants. Similarly, the study by Unterrainer et al. (66), which applied the Inventory of Personality Organization [IPO-16; (57)] to asses deficits in personality organization, revealed increased primitive defense mechanisms in SUD inpatients compared to healthy controls.

### Summary of Psychoanalytic SUD Concepts

The psychoanalytic literature on the subject of SUD encompasses over 100 years and several paradigmatic shifts regarding attempts at understanding this disorder. Early analytics tried to frame SUD in the context of drive, oral regression, and perversion, while later theoreticians increasingly emphasized the importance of underlying object relations and ego structural deficits. However, most attempts at creating an etiological model of SUD are based on retrospective observations gained in qualitative case studies, which are prone to overgeneralizations of biographical findings and lack sufficient quantitative-empirical research validating the developed theories (68). Moreover, several large-scale quantitative longitudinal studies were unable to confirm central assumptions of the drive theory of SUD, like increased orality in childhood and homosexual tendencies predicting alcoholism in adulthood (69–71). In addition, the standard psychoanalytic treatment of SUD proved to be rather unsuccessful (72), which brought about the necessity of adapted psychotherapeutic strategies.

Furthermore, for most of the 20th century psychoanalysis ignored significant advances in neurobiology, leading to an increasing distance from natural science and academic psychology (73). However, this crisis of psychoanalytic theory fostered the development of the field of neuropsychoanalysis, which aimed at linking psychoanalysis with neuroscience and biological psychiatry.

### Affective Neuroscience and Neuropsychoanalytic Approaches Towards Substance Use Disorder

At the beginning of his career, Freud (74) was already trying to develop a psychology grounded in neuroscience which, however, was aborted due to a lack of sufficient research methods. Nevertheless, the project to embed psychoanalysis within a neuroscientific framework had a resurgence in the late 20^th^ century. This paradigmatic shift aimed on the one hand to counteract the increasing scientific deadlock and isolation of psychoanalytic theory and, on the other hand, to overcome the reductionist approach of biological psychiatry and neuroscience (75).

**Figure 1:**
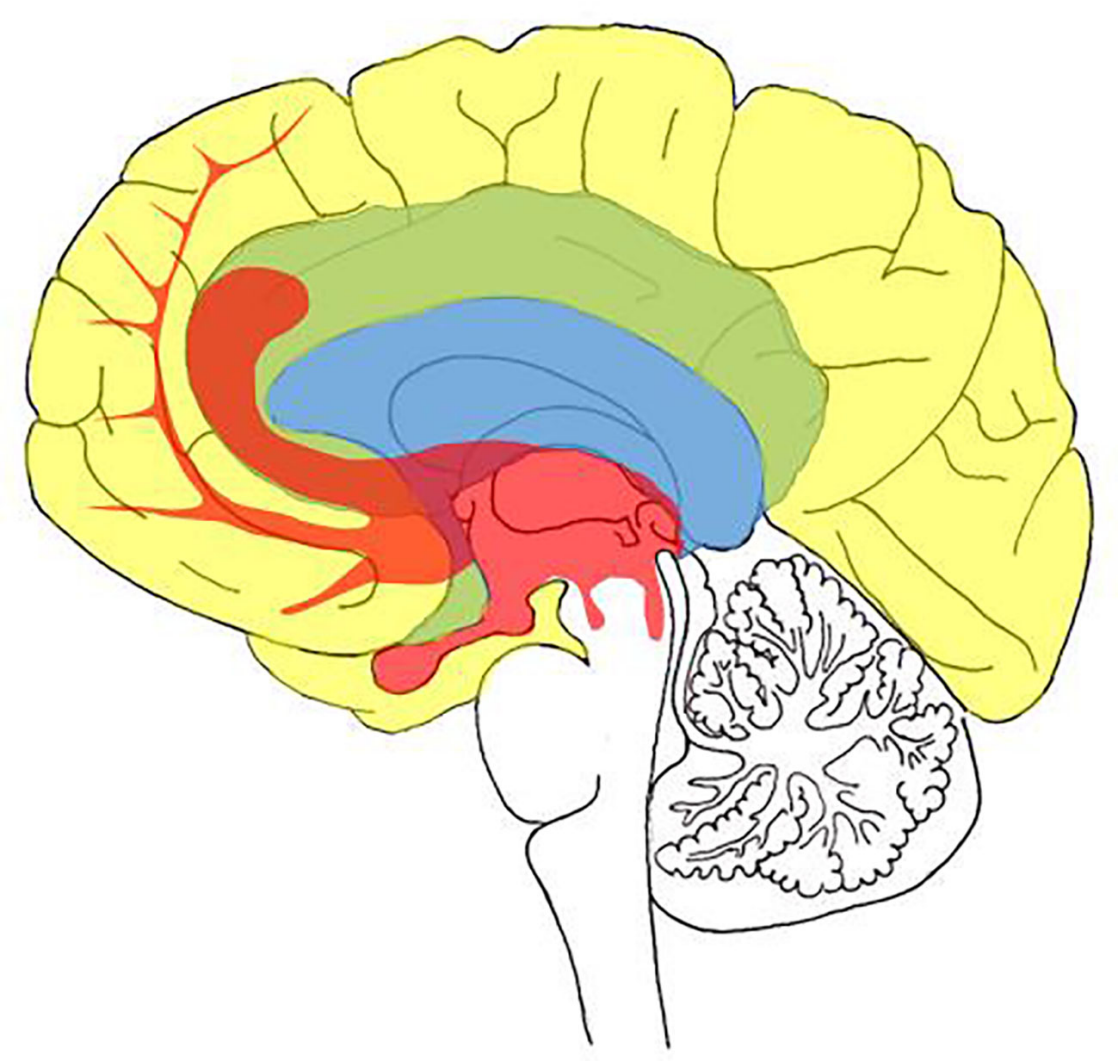
Schematic visualization of the BrainMind as proposed by Panksepp (78): Primary process areas (red); secondary process areas (blue); tertiary process areas (yellow) processes.

This approach is significantly influenced by the findings of Affective Neuroscience (AN), decisively developed by Jaak Panksepp (76), who emphasized an evolutionary perspective and affective cross-species similarities. His fusion of psychoanalysis and AN, labeled as neuropsychoanalysis, proposes a monistic relationship between mind and brain, which is expressed in the term *BrainMind* (77). The BrainMind is conceptualized as an interdependent and multi-layered dynamic structure comprised of primary, secondary and tertiary processes (see **Figure 1**) (77, 79).

As outlined in **Figure 1**, primary processes are comprised of largely subcortically (upper brainstem to the septal area) rooted networks including: (a) Homeostatic internal bodily drives like hunger or thirst; (b) Sensory affects like pleasurable taste or disgust; (c) Primary-process emotions, currently identified as networks for SEEKING, ANGER, LUST, FEAR, PANIC/GRIEF, CARE, and PLAY. These primary processes serve as the primary motivational system of behavior.

Secondary processes correspond with operations, linked to cognition and exteroceptive representations, which are largely based in the basal ganglia and upper limbic structures and mediate memory and the ability to learn behavior *via* classical, instrumental, and operant conditioning. Secondary processes include largely unconscious behavioral traits, like personality structure, attachment patterns, and object relations.

Tertiary processes are linked to more abstract cognitive operations and reflective awareness. These processes are predominantly neocortically and language based and summarize a broad spectrum of complex memory, cognitive, and executive functions like mentalization [meaning the ability to understand behaviors and mental states of oneself and others (80)], identity narratives, mindfulness, and spirituality. In each case, the different layers are reciprocally linked and therefore influence each other *via* bottom-up processes and top-down regulatory control.

At present, AN and neuropsychoanalytic researchers categorize seven primary emotion networks, which ascend from the PeriAqueductal Gray (PAG) into the limbic forebrain (77).

Four of those systems have evolutionary reptilian roots (81). These comprise SEEKING, which mediates appetitive foraging; FEAR, mediating freeze and flight behavior; LUST, mediating sexual and consummatory pleasure; and ANGER, mediating aggressive attack behaviors.

Furthermore, three basic emotion circuits specifically manifest in evolutionarily higher species like certain birds and mammalians. These systems consist of PANIC/GRIEF or SADNESS, which mediates separation distress; CARE, mediating nurturing behavior; and PLAY, mediating rough and tumble playing behavior (76, 77, 81).

### The Role of Primary Emotions in SUD Development

All seven primary emotion networks are assumed to have relationships with a variety of psychiatric disorders (82). Regarding the etiology of SUD, the theory of neuropsychoanalysis focuses on the significance of SEEKING and PANIC/GRIEF (82–85).

#### SEEKING and SUD

**Figure 2:**
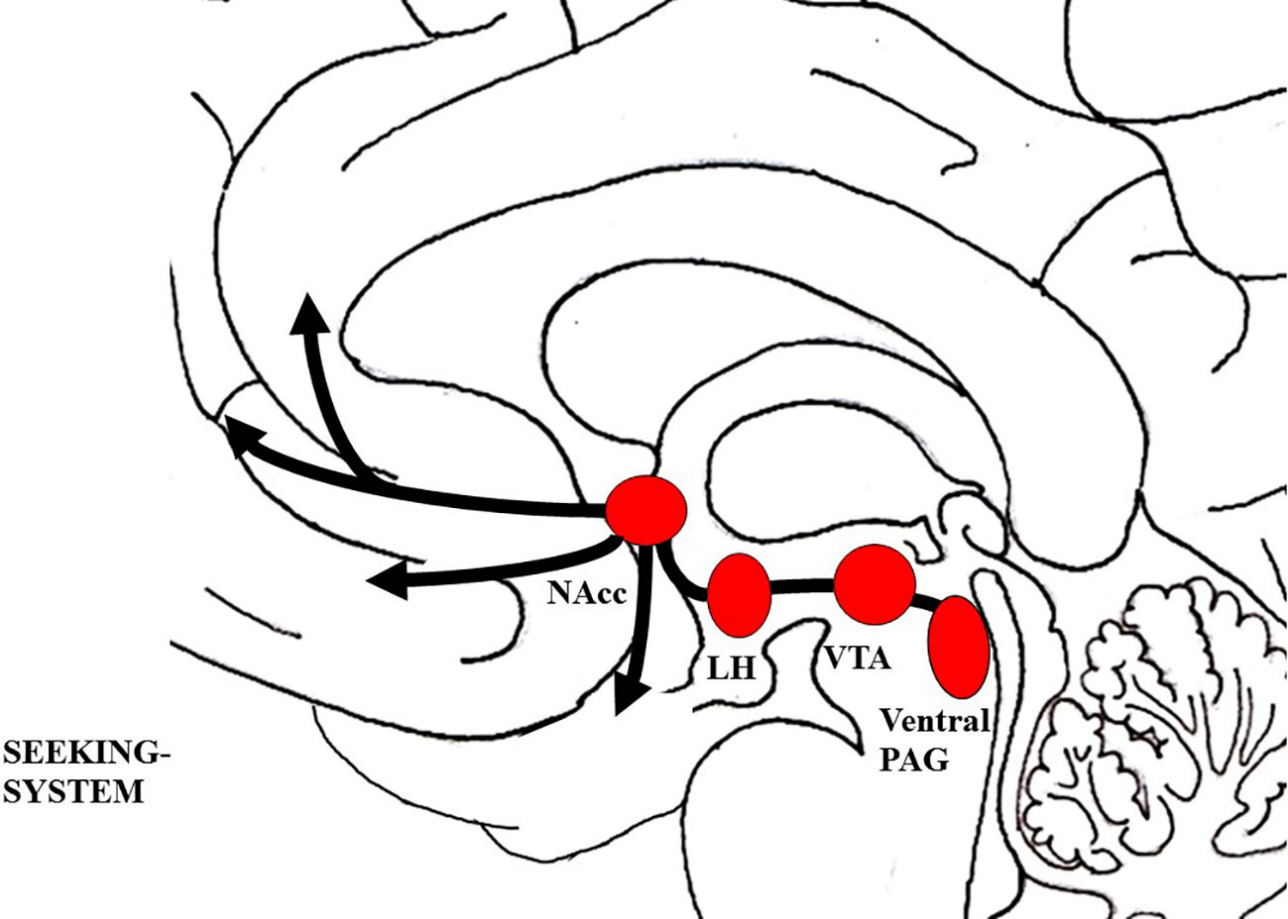
Schematic visualization of the SEEKING system; The figure was drawn by J. Fuchshuber, based on theoretical concepts by Panksepp (78). LH, Lateral hypothalamus; NAcc, Nucleus accumbens; PAG, Periaqueductal gray; VTA, Ventral tegmental area.

Neuroanatomically, the SEEKING system largely corresponds to the medial forebrain bundle (MFB) (see **Figure 2**), and includes a complex network between the dorsal PAG, Ventral Tegmental Area (VTA), lateral hypothalamus, NAcc (which projects towards the amygdala), frontal cortex areas, and the Anterior Cingulate Cortex (ACC) (78, 81). Neurochemically, the SEEKING system is modulated largely by dopamine projected by the VTA, but also by descending Gamma-AminoButyric Acid (GABA) and glutamate systems and other ascending catecholamine systems (like norepinephrine and serotonin) as well as neuropeptides projected from source neurons within the lateral hypothalamus (86, 87).

Largely in consensus with Berridge (88, 89), AN theorists suggest that SUD is linked to pathological shifts within the SEEKING/wanting system. During the development of a SUD, SEEKING is increasingly and finally primarily activated in association with substance related appetitive memories, substance consumption, and the desire to alleviate negative affective states (83, 85, 90). Moreover, it is emphasized that several lines of evidence support the hypothesis that individuals predisposed to SUD might be vulnerable to the development of this disorder due to specific psychological and neural predispositions like depressiveness or hyperactivity of the brain stress system. Consequently, this might foster the restructuring of SEEKING towards substances of abuse or addictive behaviors like gambling (83). In correspondence to this, animals predisposed to SUD showed traits associated with the concept of “novelty seekers” or “sensation seekers” (91–93), like increased locomotion and explorative behavior in novel environments as well as a higher preference for novel environments (83, 94). Furthermore, animal models indicate that vulnerability to SUD is linked to increased approach behavior towards cues previously associated with rewards, in contrast to approach behavior towards the reward itself (95, 96). This enhanced conditioning power of environmental stimuli is described by the “incentive salience hypothesis” by Berridge and Robinson (97). In this model, it is proposed that dopamine activity predicts the reward gained from a specific object, which is called “incentive”. In turn, this motivates the organism to approach this object, which is summarized in the term “salience”. Regarding SUD, the drug induced increased dopamine release might lead to an overly strong incentive of the drug and associated environmental cues, which promotes their excessive salience (88, 97, 98).

This is underlined by findings indicating low basal dopamine levels and dopamine D2 receptor availability in humans predisposed to SUD and with a history of SUD (99–101). In line with this, AN-researchers argue that a predisposition towards SUD is linked to a general hypoactivity of the SEEKING/MesoLimbic-DopAminergic (ML-DA) network (83–85). In turn, this is linked to a diminished capacity to seek rewards in the external world. This fosters a gradual development in which the individual learns that only excessive surges of dopamine D2 driven excitation, as triggered by addictive drugs, allows for the achievement of pleasurable objects in the external world (84). Therefore, it is proposed that the object of SUD is not the addictive substance, but rather the *possibility* of actual biological, social, or sexual rewards, facilitated by the effect of the substance (83–85). In correspondence to this, the SEEKING network would play a relevant etiological role especially regarding SUDs that involve stimulating drugs, like cocaine and amphetamines (84, 85). This is further emphasized by recent results indicating that striatal dopamine receptor availability is diminished in individuals suffering from stimulant and alcohol addiction, but not in patients suffering from opiate or cannabis addiction (102).

Another line of evidence which associates SEEKING with SUD is based on research focused on the so called “drug dreams” phenomenon found in SUD patients (103). With regard to Solm’s (104, 105) neuropsychoanalytic dream theory—identifying the SEEKING system as essential for the generation of dreams—several studies observed that SUD patients frequently report dreams related to their craving of the drug they are addicted to (106–110). Usually, these dreams contain episodes in which subjects seek for drugs, and either use them or attempt to use them. It was also observed that these dreams are associated with increased drug-craving (106, 107, 111, 112). Hence, drug dreams are interpreted as hallucinatory wish fulfillment of drug cravings mediated by an upregulated SEEKING system (108).

### PLEASURE/LUST, PANIC/GRIEF, and SUD

What is more, AN-theory emphasizes the significance of the PANIC/GRIEF and PLEASURE/LUST systems regarding the development of SUD (84, 85, 113).

**Figure 3:**
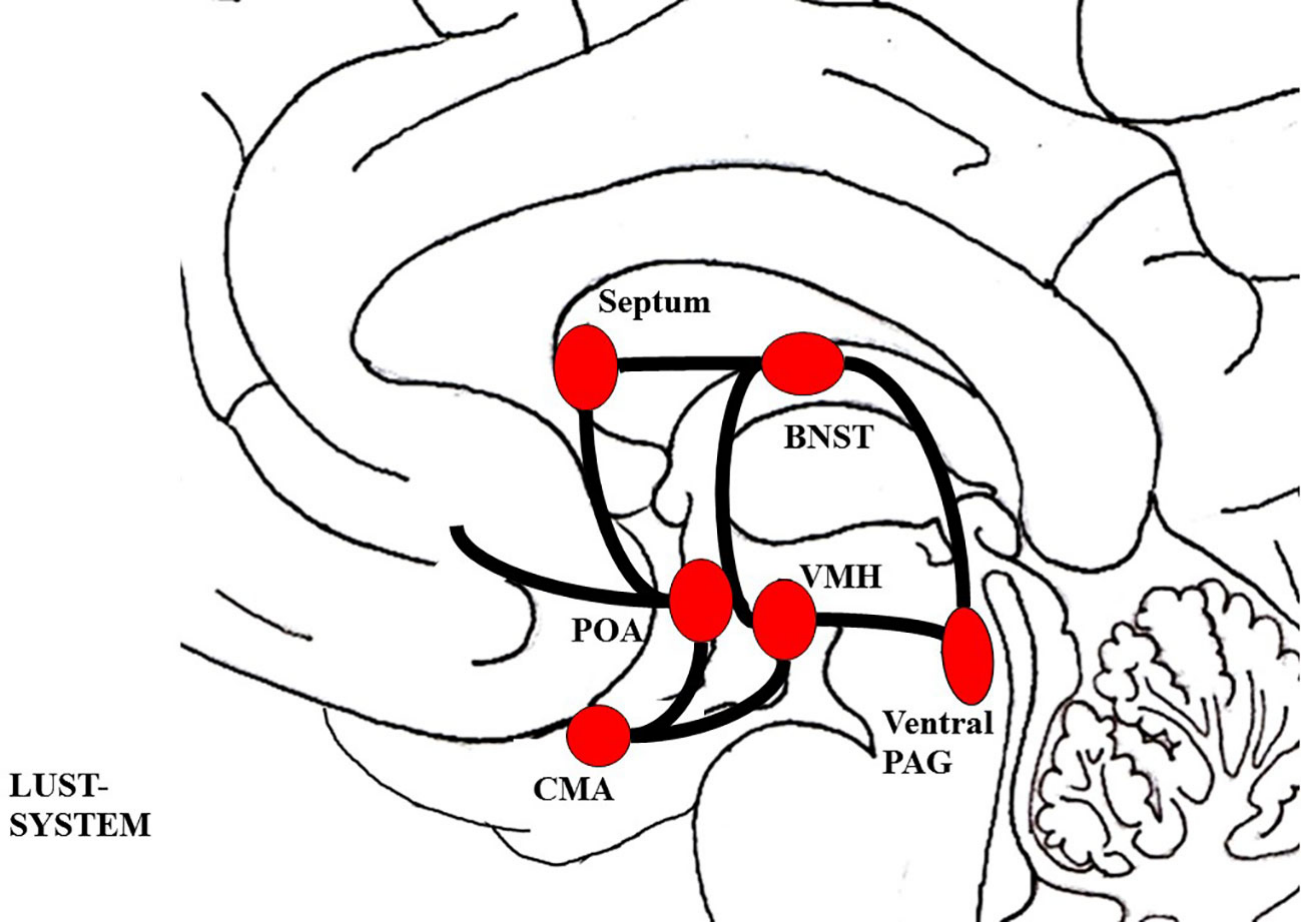
Schematic visualization of the LUST system; The figure was drawn by J. Fuchshuber, based on theoretical concepts by Panksepp (78) and Solms and Turnbull (114). BNST, Bed nucleus of the stria terminalis; CMA, Cortico-medial amygdala; POA, Preoptic area; PAG, Periaqueductal gray; VMH, Ventromedial hypothalamus.

The LUST system, which is linked to pleasure, sexual urges, and gratification, is reciprocally connected to the SEEKING network. Its activation diminishes SEEKING driven appetence behavior and triggers behaviors and feelings of satisfaction (114). Regarding its neuroarchitecture, the LUST network is not yet fully understood in humans and the theory behind its structural connectivity is inconsistent across different authors. Inferred from animal models it consists of a complex group of structures, descending from the hypothalamus to the posterior parts of the midbrain (see **Figure 3**) (81). Most authors agree that the LUST/sexuality system includes the Bed Nucleus of the Stria Terminalis (BNST), the central tegmental field, the preoptic area and the ventromedial hypothalamus, the NAcc shell, septum area, and the ventral PAG (77, 81, 115–117). Neurochemically, the LUST system is largely controlled by endorphins acting on mu-, delta-, and kappa-opioid receptors in the NAcc shell,

And hormones like vasopressin, testosterone, and oxytocin (77, 114, 116).

**Figure 4:**
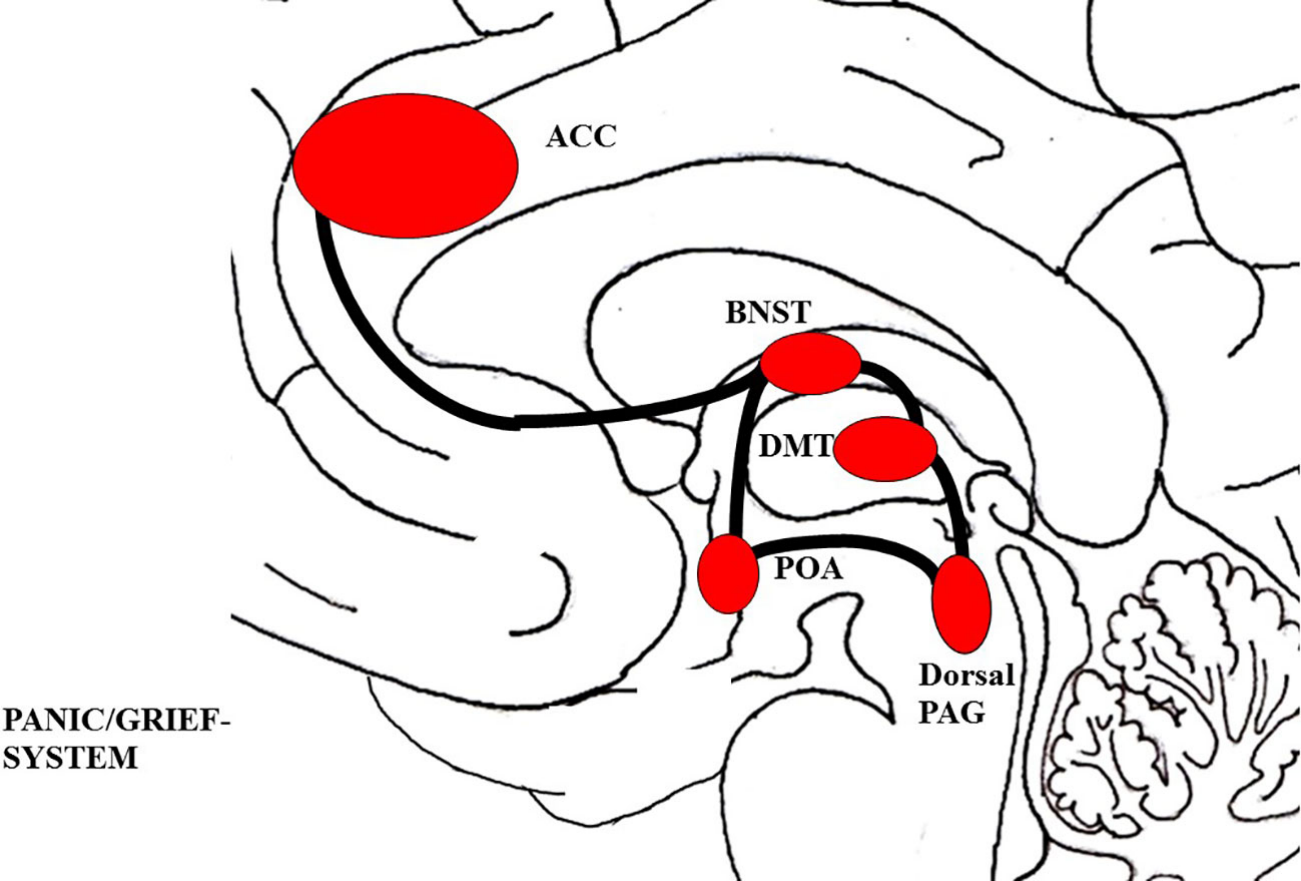
Schematic visualization of the PANIC/GRIEF system; The figure was drawn by J. Fuchshuber, based on theoretical concepts by Panksepp (78) and Solms and Turnbull (114). ACC, Anterior cingulate cortex; BNST, Bed nucleus of the stria terminalis; DMT, Dorsomedial Thalamus; POA, Preoptic area; PAG, Periaqueductal gray.

As shown in **Figure 4**, the PANIC/GRIEF system includes connections between the ACC, the BNST, the preoptic area, and the dorsomedial thalamus which descend to the PAG (see **Figure 6**) (77, 78). This system is predominantly controlled by endogenous mu-, delta-, and kappa-opioid-receptors. In correspondence to this, endogenous mu and delta opioid receptor ligands (like enkephalins and endorphins) deactivate the PANIC/GRIEF system, while kappa-opioid ligands (like dynorphins) increase PANIC/GRIEF activity (77, 119). Moreover, this system is deactivated by the hormones oxytocin and prolactin and activated by Corticotropin-releasing hormone (CRF) and the neurotransmitter glutamate (78).

Neuropsychoanalytic authors propose that it is not the dopamine surges linked to the artificially excited SEEKING system but the feeling of reward itself, mediated in large part by the primarily opioid controlled PLEASURE/LUST and PANIC/GRIEF systems that are the dominant object of SUD. Moreover, it is proposed that there might be a clinically useful differentiation between SUDs primarily involving substances which stimulate the SEEKING system (“upper”) and SUDs primarily involving substances which stimulate the PLEASURE/LUST and PANIC/GRIEF system (“downer”), with the latter being the clinically more severe disorder (84, 85).

What is more, the neuro-architecture of attachment in mammalians, predominantly mediated by the PANIC/GRIEF system and SUD, share remarkable resemblances, which are paralleled by a compelling overlap in the behavioral aspects of social dependence and SUD (76, 85, 120–122). Mutual neurochemical sites of action and change regarding attachment and SUD development include dopamine D1 and D2 receptors, μ-, Δ-, and κ-opioid receptors, and CRF (120). On a behavioral level similarities between SUD/withdrawal attachment/loss include: Drug dependence – social bonding; drug tolerance – alienation; and drug withdrawal – separation distress (76, 84, 85). Moreover, the phenomenology of opioid withdrawal show notable resemblances to separation distress, comprising somatic and mental pain, crying, loss of appetite, depression, insomnia, and agitation (76, 84, 85). In this context, SUD is seen as a dysfunctional attempt to compensate for overwhelming feelings of isolation, loss, and sadness mediated by an overactive PANIC/GRIEF system. Hence, the abuse of μ- and Δ-opioid receptor agonists might especially act as a replacement for secure love objects, substituting the warmth and comfort usually experienced in close relationships.

### Despair as a Common Denominator of Depression and SUD

In correspondence to the proposed relation between SUDs, decreased SEEKING, and increased PANIC/GRIEF, the AN-framework sheds new light on the link between depression and SUD, already emphasized by Rado (11, 23). AN-theory frames depression as an evolutionarily conserved mechanism in which the hyperactive PANIC/GRIEF system shuts down the acute panic or protest phase of separation distress and triggers a state of *despair* which is characterized by sustained overactive PANIC/GRIEF and discontinuation of the SEEKING system, experienced as intense dysphoria (119). This mechanism seems to be mediated by increased dynorphin activity and reduced dopamine transmission within the ML-DA (123, 124). Similarly, SUD development is driven by the antagonist affective process triggered by the dysphoria of diminished SEEKING resources, either because of sustained artificial over-stimulation through drug consumption, a premorbid disposition towards SEEKING hypoactivity, or chronic hyperactivity of the PANIC/GRIEF network (85). Therefore, despair (increased PANIC/GRIEF and decreased SEEKING) is proposed as the common affective core of both disorders. However, individuals suffering from SUD self-medicate this painful affective state *via* the consumption of psychoactive substances. The plausibility of this assumption is underscored by recent progress in the neuroscientific research emphasizing the significance of dopamine and opioid systems in the etiology and treatment of depression and SUD (125–127) and the firmly established correlation between both disorders (128, 129). Furthermore, there has been a long tradition linking both SUD and depression to loss, mourning, and insecure attachment (8, 51, 130, 131).

Until recently, the influence of other primary emotion systems in the emergence of SUD cycles has been largely neglected in AN-theory and research. Nonetheless, the results of Unterrainer et al. (132) suggested increased SADNESS, FEAR, and ANGER dispositions in patients diagnosed with polytoxicomania compared to healthy controls. In addition, little is known about the role of PLAY and CARE in SUD etiology. Regarding the neurochemistry of PLAY, which partly relies on the endogenous cannabinoid system (77), it might be conceivable that PLAY is involved in cannabis SUD, however this hypothesis lacks empirical support (132). Similarly, there is no data suggesting the significance of CARE in SUD development in humans thus far. However, animal research has shown that lactating dams exhibit decreased activity in the ML-DA compared to virgin females if they had been exposed to cocaine (133). Therefore, the question remains if SUD should be understood as a self-medication strategy against negative affects in general, as proposed by authors of ego-psychology [e.g. (42)], rather than a distinct coping strategy against hyperactive PANIC/GRIEF and hypoactive SEEKING activity as suggested in AN-theory.

### Trauma, Attachment, and SUD

In psychodynamic theory, the experience of childhood trauma has been assumed to play a crucial role in the etiology of psychiatric disorders since its beginnings at the end of the nineteenth century (134, 135). In a psychoanalytic context, trauma is defined as an event so intense and overwhelming that it is impossible for the subject to react functionally to it and results in an enduring pathogenic effect (53). Empirically, childhood trauma is frequently measured by retrospective assessments of emotional, physical, and sexual abuse, as well as emotional and physical neglect and deprivation (136, 137).

Meanwhile, contemporary research has gathered a considerable amount of evidence linking traumatic environments in childhood to a wide range of adult psychopathology (138). In correspondence to this, a recent review by Teicher and Samson (139) suggested that childhood trauma is substantially associated with structural changes in a number of brain regions linked with the processing and modulation of emotions. Specifically, this includes the anterior cingulate, dorsal lateral prefrontal and orbitofrontal cortex, the corpus callosum, and the hippocampus. Furthermore, childhood trauma is associated with increased amygdala response to emotional cues and conflict processing as well as a reduced striatal response to anticipated rewards. Hence, converging evidence indicates that the link between childhood trauma and adult psychopathology might be conveyed by disturbances in the neurobiological development linked to cognitive control and emotion regulation (140–142).

In correspondence to this, the development of secure attachment—which in line with AN-theory might be seen as a secondary order process (77)—has been linked to the development of emotional functioning (143). According to attachment theory, the emergence of emotion regulation relies on the early nonverbal communication between infant and caregiver (144, 145). Ideally, primary caregivers perceive the nonverbal affective expressions of the infant and co-regulate these through symbolic mirroring and by providing physical and verbal comfort. This process supports the infant in tolerating its intense and unsymbolized affects. The repetition of this process leads to a gradual internalization of positive inner working models of the self and others. The positive inner working models serve as an internalized secure base which supports the individual in regulating emotions in a functional and relatively autonomous way, and enables him/her to explore the external world on his/her own (146). Moreover, secure attachment patterns facilitate the formation of stable and functional relationships, allowing the individual to additionally regulate emotions with the help of others (121). In contrast, internalized traumatic early experiences promote the development of corresponding negative inner working models and insecure attachment patterns that hamper the functional regulation of emotions and the formation of stable relationships (80, 145, 147, 148).

Due to the relationship between insecure attachment and affective dysregulation, insecure attachment has been repeatedly linked to the development of addiction disorders (149, 150). Largely in line with psychoanalytic object relations theory and ego-psychology, substance abuse is seen as a chemical affect regulation strategy, substituting a secure attachment figure and acting as an artificial “secure base” for the consumer (121, 151). Initially, this has a stabilizing effect on the self and its affect regulation capabilities. However, ultimately substance abuse further weakens attachment abilities and affect regulation, which triggers the vicious addiction circle of increased substance abuse, gradually leading to a complete loss of control. The assumed association between SUD and insecure attachment is well supported by a multitude of empirical studies applying psychometric attachment measures (149, 150, 152).

## Summary of the Neuroscientifically Informed Psychodynamic Approach Towards SUD Etiology

In summary, all psychodynamic theories reviewed above share a common etiological structure explaining the emergence of SUD and other psychiatric disorders (1). Therefore, all psychodynamic approaches can be summarized thus: (1) An interaction between biography and hereditary dispositions determine (2) the individual’s level of integration regarding his/her personality structure, which is comprised of the relationship between primary (including drives and affects), secondary, and tertiary processes (including ego and super-ego structures, object relations, and attachment working models). These developmental deficits are reflected on a neurobiological level and promote (3) a failing adaption to reality and its demands. In turn, this leads to (4) the manifestation of psychiatric disorders, which (5) in the case of maladaptation by the use of psychoactive substances leads to the development of addictive behaviors and ultimately to the manifestation of SUD (1, 41).

As outlined above, the historical development of the psychodynamic discourse on the phenomena of SUD might be described as a movement, starting from an emphasis of sexual and aggressive drives—sometimes crossing the border to moral judgment and stigmatization—to a broader and more empathetic understanding of SUD as a dysfunctional coping strategy, aimed at managing intolerable pain and suffering (153). This theoretical development is paralleled by a refinement of psychodynamic treatment strategies for subjects affected by SUD. Instead of the traditional psychoanalytic method, which is largely based on the interpretation of latent conflicts by an abstinent analyst (53), contemporary psychoanalysis stresses a more supportive, understanding, and active role of the psychotherapist (45, 154, 155). In correspondence to this, the psychodynamic therapist attempts to be perceived by the patient as a “sufficiently good” object, which might be gradually internalized. In turn, this is aimed at fostering increased interpersonal, affect regulation, and mentalization capabilities on the part of the patient (156, 157).

## Research Focused on the Relationship Between Childhood Trauma, Personality, and Substance Use Disorder

In correspondence to previous research conducted by our research group (65, 66, 132) which linked SUD to deficits in personality organization and primary emotions, we aimed at increasing the understanding of this association by including the impact of childhood trauma and applying path analytical and structural equation modeling techniques to extensive samples. For this purpose, the first study (158), tested assumptions proposed by Zellner et al. (85) and Solms et al. (84) focusing on the role of childhood trauma, despair, and personality organization in the development of SUD and depressive symptoms. (118), which was based on an extended sample of Fuchshuber et al. (158), examined the role of childhood trauma, adult attachment, and personality organization on overall primary emotion functioning in more detail. Finally, study three (159) conducted a secondary analysis of the sample described in study two (118), but focused on the relationship between primary emotions, symptoms of SUD, and mood pathology as well as gender. A brief overview of the methods and results of each study can be found in **Table 1**. Further information on the presented studies (e.g. detailed sample characteristics, statistical analyses) can be found in the respective publications (118, 158, 159) or obtained from the authors.

**Table 1:** Methods and results of studies on SUD.

Sample		Methods		Results
Study 1	n = 500	IPO-16, BSI-18, ANPS, ASSIST, CTQ	Structural equation modelling	**Correlation among latent variables:** Addictive behavior and depressive symptoms are significantly linked with more severe Childhood Trauma, increased Despair (decreased SEEKING and increased SADNESS) and Structural Deficit. **Structural equation modelling:** Influence of Childhood Trauma on Addictive Behaviors was mediated by structural deficit. Its influence on depressive symptoms was mediated by Despair (decreased SEEKING and increased SADNESS).
Study 2	n = 616	IPO-16, ANPS, ASSIST, CTQ	Path analysis	**General path analysis:** Childhood trauma predicted deficits in personality organization and insecure attachment. Increased structural deficit was associated with increased ANGER. Increased adult attachment security predicted primary emotion dispositions in general. **Mediation analysis:** The influence of childhood trauma on ANGER was mediated by structural deficit and comfort with depending on others. Its influence on other basic emotion dispositions was mediated by adult attachment security.
Study 3	n = 616	ANPS, ASSIST, BSI-18	Path analysis	**General path analysis:** Substance abuse was associated SADNESS and ANGER. Anxiety was linked to SADNESS, FEAR and PLAY. Somatization was associated with SADNESS, and PLAY. Depression was linked to SADNESS, SEEKING, FEAR and PLAY **Multi-group analysis:** Comparison between groups (female vs. male; “healthy” vs. participants reporting a lifetime psychiatric diagnosis) revealed no significant difference between paths.

AAS, Adult Attachment Scales; ANPS, Affective Neuroscience Personality Scales; ASSIST, The Alcohol, Smoking, and Substance Involvement Screening Test; BSI-18, Brief Symptom Inventory; CTQ, Childhood Trauma Questionnaire; IPO-16, Inventory of Personality Organization.

### Study 1

With regard to (84, 85), we aimed at exploring the common neuropsychodynamic structure of SUD and depressive symptoms. Both disorders, which are two of the most frequently observed psychiatric disorders (160) are known to be considerably correlated (128, 129). As described above, AN-theory proposes a common primary affective core for both disorders, which is characterized by dysregulations within the SEEKING and SADNESS or PANIC/GRIEF system (84, 85). In correspondence to this, patients suffering from SUD and patients suffering from depression were expected to report increased SADNESS scores and decreased SEEKING scores. Therefore, the investigated model included the latent variable “Despair”, which was composed of low SEEKING and high SADNESS. Furthermore, based on previous research (58, 65, 66), we expected significant associations between both disorders and impairments in personality organization. Moreover, with regard to a long line of research linking childhood trauma to impaired personality structure, increased negative affect, and adult psychopathology (134, 135, 138, 140, 145, 161), we assumed a mediating role for despair and personality organization in the relationship between childhood trauma, SUD, and depressive symptoms.

### Methods and Results

This study investigated a sample of young adults aged between 18 and 39 years (M = 26 years; SD = 5.51), which included 500 (63.2% female) German-speaking participants. 37.4% of the investigated participants reported a lifetime diagnosis with a psychiatric disorder. A majority of these participants declared a diagnosis with depression (n = 129; 69%) and 9% reported a diagnosed form of SUD. All participants completed the German version of the *Affective Neuroscience Personality Scales* [ANPS; (162); German version by Reuter and Hennig (2014; see (163)], the *16-Item Inventory of Personality Organization* [IPO-16; (61)], the *Childhood Trauma Questionnaire* [CTQ; (137); German version by (164)], the “Depression” subscale of the *Brief Symptom Inventory* [BSI-18; (165); German version: (166)], and the *Alcohol, Smoking and Substance Involvement Screening Test* [ASSIST; (167)].

Correlation analysis among latent variables revealed significant correlations between every investigated factor (p < 0.001), which were in line with previous assumptions.

**Figure 5:**
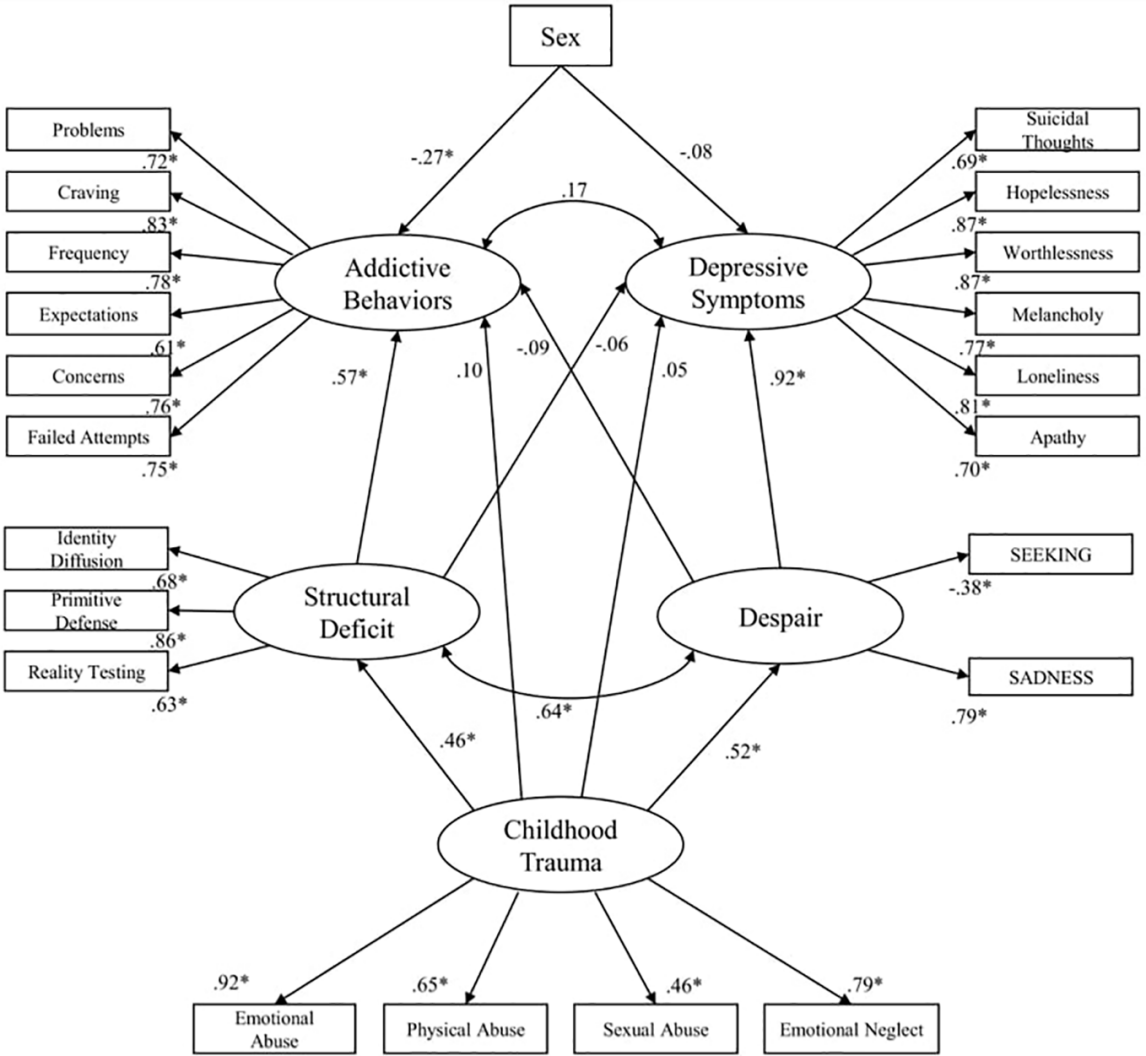
Initial standardized solution for the structural equation model. *p < .001; Sex: Female = 1; Male =0 (158).

The initial standardized solution for the structural equation modeling (see **Figure 5**) exhibited an acceptable model fit: RMSEA = 0.06 (90% CI: 0.06, 0.07); TLI = 0.92; CFI = 0.93; AIC = 737.575. In this model, Addictive Behaviors is significantly associated with Structural Deficit (β = 0.57; p < 0.001) and male sex (β = -0.27; p < 0.001), while Despair significantly predicted Depressive Symptoms (β = 0.92; p < 0.001). Furthermore, Childhood Trauma did not show significant direct effects on either psychiatric variable but was associated with Structural Deficit (β = 0.46; p < 0.001) and Despair (β = 0.52; p < 0.001). Bootstrap analysis showed significant indirect effects of Childhood Trauma on Addictive Behaviors *via* Structural Deficit (β = 0.22; p < 0.01) and on Depressive Behaviors *via* Depressive Symptoms (β = 0.44; p < 0.01).

### Study 2

Building upon insights gained in previous research (130, 132, 158, 168), Fuchshuber et al. (118) focused on mapping the relationship between childhood trauma, adult attachment, personality organization, and primary emotion functioning in more detail. We expected the influence of childhood trauma on primary emotion functioning to be mediated by adult attachment and personality organization, as recent psychodynamic literature assumes these concepts to be influenced by early childhood relationships and, in turn, associated with affect regulation in adults (56, 168).

### Methods and Results

For this study, a community sample of 616 adults [61.9% female; extended sample of (158)], was investigated. The mean age of participants was M = 30 (SD = 9.53) and ranged between 18 and 69 years. 39.4% of the participants reported a psychiatric lifetime diagnosis. A majority (60%) of these participants were diagnosed with depression. AMOS 18.0 was used to estimate paths and fit indices of the path analytic models.

All participants completed the German version of the *Affective Neuroscience Personality Scales* [ANPS; (162); German version by Reuter and Hennig (2014; see (163)], the *16-Item Inventory of Personality Organization* [IPO-16; (61)], the *Childhood Trauma Questionnaire* [CTQ; (137); German version by (164)], and the *Adult Attachment Scale* [AAS; (169); German version: (170)].

**Figure 6:**
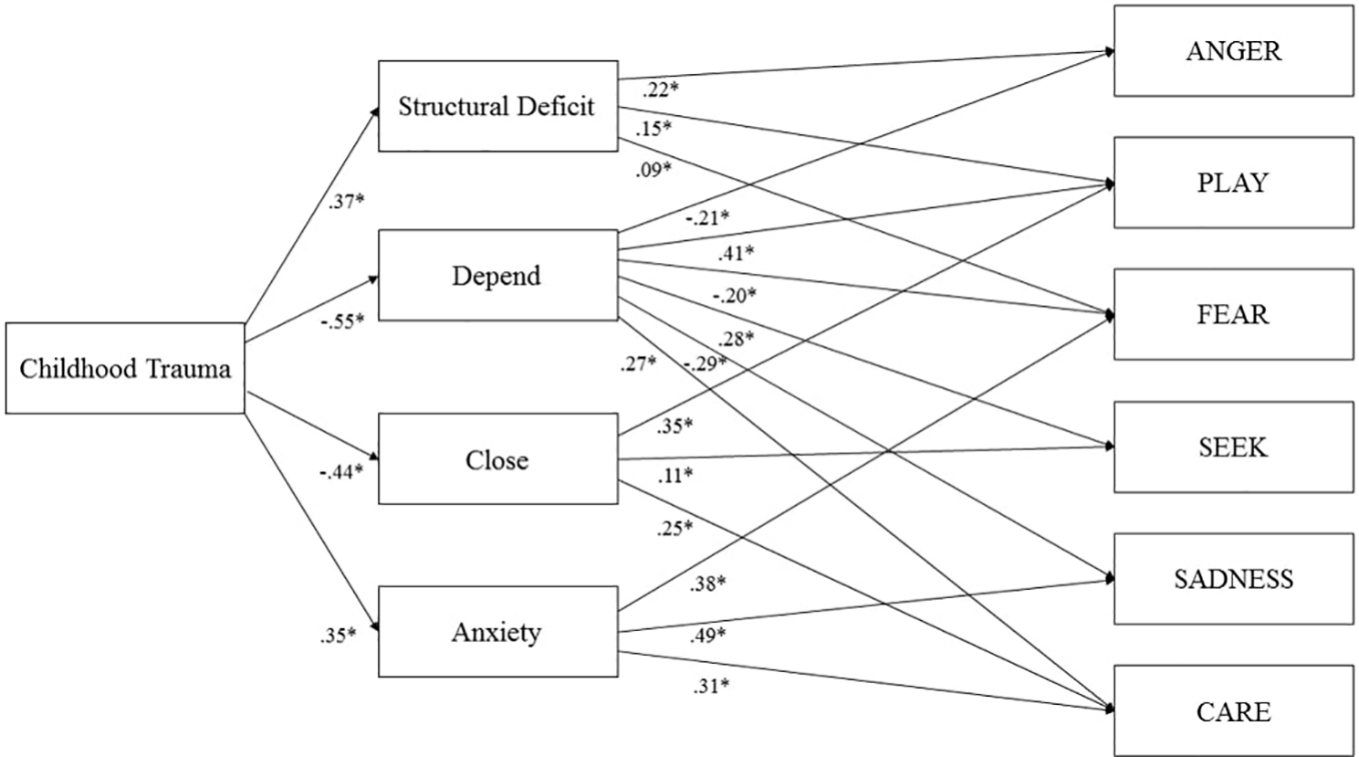
Final model of Childhood Trauma, Structural Deficit, adult attachment, and primary emotions (118). *p < .001.

The final model exhibited an excellent model fit: RMSEA = 0.03 (90% CI: 0.01, 0.05); TLI = 0.99; CFI = 1.00. As shown in **Figure 6**, this model suggested the following associations: Childhood Trauma was significantly associated with Depend (β = -0.56; p < 0.001), Close (β =-0.44; p < 0.001); Anxiety (β = 0.36; p < 0.001), and Structural Deficit (β = 0.39; p < 0.001). Structural Deficit was associated with ANGER (β = 0.21; p < 0.001) and PLAY (β = 0.12; p < 0.001). Depend was associated with SADNESS (β = -0.29; p < 0.001); ANGER (β = -0.21; p < 0.001) and SEEK (β = 0.29; p < 0.001); FEAR (β = -0.25; p < 0.001); PLAY (β = 0.41; p < 0.001) and CARE (β = 0.24; p < 0.001), while Close was associated with SEEK (β = 0.10; p < 0.02); PLAY (β = 0.33; p < 0.001); and CARE (β = 0.25; p < 0.001). Anxiety was associated with FEAR (β = 0.37; p < 0.001); CARE (β = 0.27; p < 0.001) and SADNESS (β = 0.47; p < 0.001). Finally, Structural Deficit was significantly correlated with every attachment scale (p < 0.001) and every attachment scale was significantly correlated with each other (p < 0.001).

What is more, bootstrap analysis revealed significant associations between Childhood Trauma and SEEK (β = -0.18; p < 0.01), CARE (β = -0.15; p < 0.01), FEAR (β = 0.29; p < 0.01) and SADNESS (β = 0.31; p < 0.01) which were solely mediated by adult attachment. Furthermore, the association between Childhood Trauma and PLAY (*β* = -0.31; *p* < 0.01) and ANGER (β = 0.20; p < 0.01) were mediated by Structural Deficit as well as adult attachment.

### Study 3

Based predominantly on intracranial stimulation studies in animal models, AN-theory proposes perturbations in subcortical affective systems as a significant factor in the emergence of a variety of psychiatric disorders (77, 82). However, studies testing these hypotheses in human subjects have been sparse. To investigate these claims within a quantitative-empirical framework, the third study (159) examined the relationship between psychiatric symptoms (SUD, depression, anxiety disorder, and somatization) and different dimensions of primary emotions (SEEKING, FEAR, ANGER, SADNESS, PLAY and CARE). Furthermore, by using the multi-group path analysis technique, this study was able to test the possible moderator effects of gender and psychiatric lifetime diagnosis.

### Methods and Results

**Figure 7:**
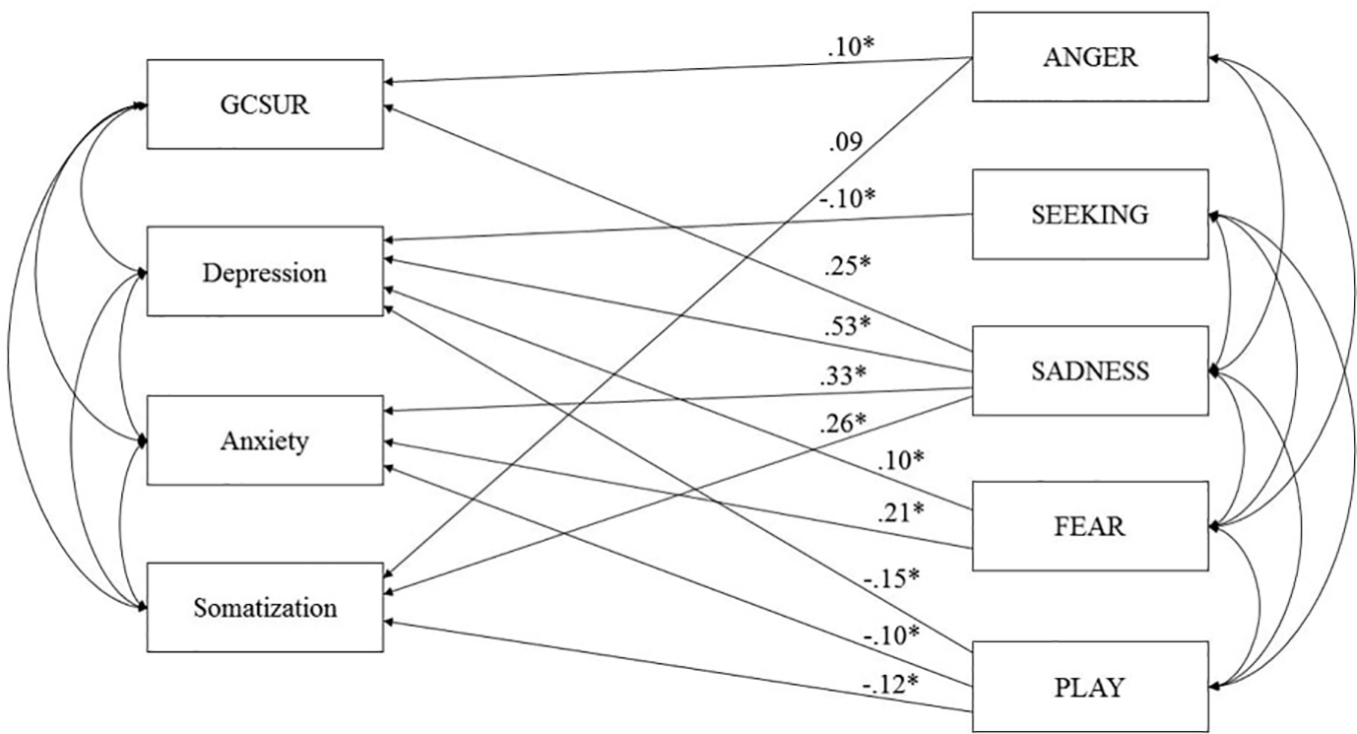
Final model of primary emotions and psychiatric symptoms controlled for Age and Sex (159). GCSUR, Global Continuum of Substance Use Risk; *p < .001; curved arrows indicate significant correlations (p < .001).

The final model showed an excellent fit: RMSEA = 0.05 (90% CI: 0.03, 0.08); TLI = 0.97; CFI = 0.99 AIC = 159.74, and suggested the following associations (see **Figure 7**): Global Continuum of Substance Risk was associated with SADNESS (β = .25), ANGER (β = .10) male sex (β = -.25). Depressive Symptoms were associated with increased FEAR (β = .10) and SADNESS (β = .53), and decreased dispositions to SEEKING (β = -.10) and PLAY (β = -.15). Anxiety Symptoms were related to increased SADNESS (β = .33), FEAR (β = .21) and decreased PLAY (β = -.10). Somatization was associated with elevated ANGER (β = .09) and SADNESS (β = .26) and diminished PLAY (β = -.12) (all p <.01). In summary, the final model was able to explain 14% of the variance of Global Continuum of Substance Use Risk, 52% of Depressive Symptoms, 32% of Anxiety symptoms, and 14% of Somatization.

Regarding possible moderation effects of psychiatric lifetime diagnosis and gender, additional multi-group analysis (female vs. male; “healthy” vs. participants reporting a lifetime psychiatric diagnosis) revealed no significant difference between paths (all p = n.s.).

## Discussion

The research presented herein investigated the complex relations between childhood trauma, (neuro-) psychodynamic personality traits, and adult psychopathology. Largely in line with previous research (171–173) and theoretical assumptions in psychoanalytic literature (38, 56, 135, 145, 146, 174), our findings underline the harmful effects of early traumatic relationships on the development of personality organization, adult attachment, and emotional functioning. This result resonates with evidence gathered by Schimmenti and Caretti (172) and Granieri et al. (173) indicating that the effects of childhood trauma on adult psychopathology are partially mediated by dissociation, which is conceptualized as a primitive defense mechanism related to splitting (58, 175). In correspondence to this, our results expand this conceptualization by suggesting that deficits in emotional functioning and personality organization fully mediate the effect of childhood trauma in the emergence of SUD and depression in adults. Furthermore, results of the studies presented above (118, 158, 159), echo previous research, which suggested significant associations between personality organization, attachment, primary emotions, and SUD (65, 66, 85, 132, 149). On a neuroscientific level, this line of evidence is paralleled by recent findings linking childhood trauma to disturbances of neurobiological structures related to emotion regulation. As outlined above, this includes the anterior cingulate, dorsal lateral prefrontal and orbitofrontal cortex, the corpus callosum and the hippocampus (139), impairments in white matter fiber tracts, specifically in the cingulum and the superior longitudinal fasciculus (176) and chronic hyperactivity of the corticotropin-releasing factor systems (140). Moreover, very recent evidence suggests detrimental effects of childhood trauma specifically affect subfields of the hippocampus which are associated with emotion regulation like the bilateral presubiculum and subiculum (177).

With regard to study one (158), our results indicated that the underlying factors—primary emotions and personality organization—derogate the correlation between symptoms of depression and SUD. This is consistent with the assumptions made in AN-theory that assume a common etiological core for both disorders (84, 85). Therefore, it seems plausible that these underlying factors are the driving forces of the often-observed comorbidity of SUDs and depression. However, our findings suggest that Despair very precisely predicts depression, while its influence on addictive behaviors is diminished by deficits in personality organization. Therefore, addictive behaviors might be seen as a compensative strategy against gaps within a corrosive personality structure as proposed by authors linked to ego-psychology (48, 178). In contrast, our results highlight the fact that the influence of personality structure on depressive symptoms is repressed by the latent factor Despair, supporting the assumption that depression emerges due to hyperactive SADNESS and hypoactive SEEKING networks (119, 179). Nevertheless, this relationship might be more complex as both studies reviewed the above gathered evidence for a substantial interrelation between personality structure and primary emotion functioning (118, 158), which is consistent with basic assumptions of AN-theory (77, 79).

In correspondence to this, our second study (118) was able to gather new insights regarding the relationship between attachment security, personality organization, and primary emotion functioning. If computed in a single model, our results suggest that Kernberg’s (59) concept of personality organization, which is linked to object relations theory, predominantly predicts ANGER, while adult attachment attitude is related to emotional functioning in general. With regards to our results, it might be concluded that deficits in personality organization and insecure attachment foster increased negative primary emotion dispositions (ANGER, FEAR, and SADNESS), while secure attachment plays an important role in increased positive primary emotion dispositions (SEEKING, PLAY, and CARE). This is with the exception of “anxiety of being rejected”, which predicted increased CARE. This finding corresponds to the relationship between this concept and the preoccupied or insecure ambivalent attachment style, linked to exuberant clinging to attachment figures in relevant literature (180, 181). In conclusion, our results indicate empirical support for basic assumptions of object relations and attachment theory, assuming internalized object relations and attachment working models as crucial for the functional integration of affects (56, 62, 143, 168, 182).

Interestingly, the finding that compared to adult attachment, deficient personality organization is substantially related to increased ANGER, reflects theoretical differences between Kernberg’s (58) conceptualization of object relations theory and attachment theory. While his theoretical assumptions, which are strongly influenced by Kleinian theory (175), emphasize the function of personality organization in the integration and neutralization of aggressive affects, aggression has considerably less significance in attachment theory (146).

What is more, in the study Fuchshuber et al. (118) we were able to shed new light on the relationship between adult attachment and personality organization. Thereby, results of the correlation analysis corresponded to previous research suggesting substantial links between both concepts (65, 146, 183, 184). More specifically, the strength of the correlations between structural deficits and attachment security scales ranged from negative medium correlations with “Comfort with Dependence” to large positive correlations with “Anxiety of being Rejected” (185), reflecting conceptual similarities of personality organization and attachment security.

Study three (159) further investigated the relationship between psychiatric disorders and primary emotions. In contrast to study one, which followed a confirmatory approach focusing on the role of despair (85), study three used path analysis to investigate the associations between SUD, mood disorders, and all primary emotion dimensions in a more exploratory manner. The results indicate that SUD symptoms are linked to increased SADNESS and, to a lesser extent, to increased ANGER. This finding echoes recent results by Unterrainer et al. (132) suggesting increased FEAR, SADNESS, and ANGER dispositions in SUD patients. However, regarding the overall explained variance, SUD might be less related to primary emotions than previously expected. This is especially the case for SEEKING which—in line with Unterrainer et al. (132)—was not associated with SUD. While contradicting evidence from neuroscientific research (83, 90, 127), this finding might be the result of conceptual differences between functional aspects of the ML-DA or SEEKING system and the trait like disposition towards SEEKING assessed by the ANPS. Regarding its role in reinforcement learning, the ML-DA/SEEKING network seems significant the etiology of SUD, yet, this might not be reflected in decreased SEEKING personality traits. Moreover, our results were collected within a cross-sectional study, therefore it is impossible to infer causal conclusions based on our findings. Hence, it is plausible that many forms of SUD can be framed as dysfunctional coping strategies against diminished SEEKING network activity as outlined by Solms (84) and Zellner et al. (85). But, owing to the cross-sectional study design, we might have been unable to detect this association, as the abuse of drugs may have artificially increased the SEEKING disposition of participants (77). Thus, to sufficiently investigate the relationship between SEEKING and SUD, it will be necessary to conduct longitudinal studies assessing SEEKING dispositions before the onset of problematic substance use. By contrast, our findings highlight the role of SADNESS and ANGER in SUD. In line with the results of Unterrainer et al. (132), this partially supports assumptions of neuropsychoanalysis (84, 85) and also reaffirms early observations of object relations theory underscoring role of aggression in SUD etiology (14, 51). This observation further supports the notion of substance abuse as a function of artificial affect regulation. By taking drugs the addicted individual tries to seal gaps in a deficient personality structure (158), which is linked to increased negative affects (56, 57, 118). Specifically, addictive behaviors seem to be associated with increased feelings of loneliness and isolation in addition to feelings of rage and aggression, which are experienced as intensely unpleasurable and ultimately overwhelming (76, 77). The observed significant relationship between SUD and SADNESS further highlights the conceptualization of SUD as attachment disorder, specifically linked to dysregulations within the endogenous opioid system (120, 121, 186). This relationship is further underscored by recent findings which indicate that bipolar patients additionally diagnosed for alcohol use disorder (AUD) or SUD are significantly more likely to show a predominant depressive polarity in contrast to patients suffering from bipolar disorder without a comorbid AUD or SUD diagnosis (187).

Furthermore, the observed association between SUD and ANGER underlines theoretical considerations that relate substance abuse to auto-aggressive behavior against venomous inner self and object representations linked to traumatic childhood relationships (14, 51, 58).

What is more, our results indicate a differential role of primary emotions in the emergence of psychiatric disorders (159). Thereby, SADNESS seems to play a significant role in all assessed forms of mood disorders (depressive symptoms, anxiety symptoms, and somatization). However, contrasting with SUD, depressiveness was also predicted by increased FEAR and diminished PLAY and SEEKING, which is largely in line with findings from Montag et al. (163). Additionally, a similar pattern was observed for anxiety symptoms, which was linked to increased FEAR and SADNESS and decreased PLAY. Therefore, these results are in line with Panksepp’s (82) hypothesis of the relevance of the SADNESS system in anxiety disorders but also underscore his theory regarding the clinical significance of PLAY, traditionally been neglected in psychiatry (77, 78). This assumption is further reaffirmed with regards to the significant link between PLAY and somatization symptoms. This association might reflect the relationship between SADNESS and the endogenous opioid system, as a hypoactivity of μ and Δ opioid system is known to promote feelings of bodily discomfort (78, 120).

### Limitations

The present study reanalyzed the sample investigated in Fuchshuber et al. (118). Therefore, the results of our analysis should not be interpreted independently from either Fuchshuber et al. (158) and Fuchshuber et al. (118). Moreover, as there is no validated measure for the assessment of LUST presently available, we were unable to estimate the etiological relevance of this basic emotion network. Despite having a key role in neuropsychoanalytic theory, LUST was not included in the ANPS as its authors claimed that people would not be open enough to report about their sexuality (162). However, this assumption seems questionable, particularly with regard to the variety of self-report measures of sexuality already existing. Hence, future research should aim at developing a self-report measure for LUST to map the AN-framework and its role in etiology to a full extend.

In correspondence to that, the presented studies exclusively tested linear models regarding the relationship between primary and higher order processes, as well as regarding their influence on the development of SUD. However, with respect to the non-linear assumptions of AN-theory (82, 188), future studies should investigate non-linear relationships to describe the complexity of these relationships in a more naturalistic manner (189). This might be achieved in future studies by the estimation of non-recursive structural equation models in longitudinal settings.

What is more, the validity of our findings is limited due to the assessment of psychiatric symptoms by self-rating measurements, which are vulnerable to distortions towards social desirability. Furthermore, it cannot be ruled out that some psychiatric symptoms and disorders were not detected in our sample due to a lack of self-reflection abilities, especially in participants suffering from psychotic disorders or severe personality disorders (190). Hence future studies should consider applying structured clinical interviews, preferably within a clinical setting, to improve the assessment of psychiatric symptoms.

The assessment of psychodynamic parameters relied on the use of self-rating measures which reflect consciously available representations of concepts that are at least partially hypothesized as unconscious. In order to strengthen the validity of our results, future projects should include other means of data collection including qualitative and semi-structured interviews.

Moreover, it should be considered that traumatic experiences in childhood are highly susceptible to splitting, dissociation or denial, and might therefore not be detectable to a full extent (172, 191).

## Conclusion and Outlook

Despite these limitations, the studies reviewed above were able to preliminarily validate fundamental etiological assumptions proposed in psychoanalytic theory, which assume early relationships as building blocks or tuning variables of adult personality structure, ultimately determining psychopathological developments (1, 56, 174, 189).

With regard to Kernberg’s (58, 59) conceptualization of personality organization, SUD treatment should thus focus on an increased availability of mature defense mechanisms and the development of more coherent identity narratives. Improvements in these areas might enable more autonomous and functional affect regulation abilities, which might be seen as a crucial factor in stabilizing the affected individual’s process of weaning, abstinence, and rehabilitation (192, 193).

**Figure 8:**
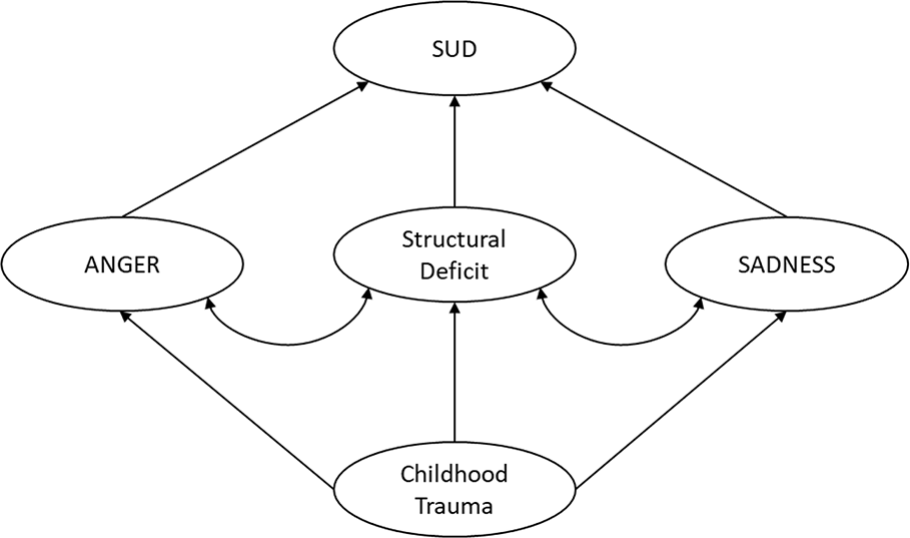
Proposed neuropsychoanalytic model of SUD etiology.

In correspondence to the role of primary emotions in SUD etiology, our research highlights the importance of increased SADNESS and ANGER. Thus, we propose the following model, which should be tested in future studies assuming the influence of childhood trauma on the development of SUD symptoms to be mediated by increased structural deficit as well as increased SADNESS and ANGER dispositions (see **Figure 8**):

In summary, it can be concluded that a neuroscientifically informed psychodynamic framework is able to contribute valuable insights regarding the underlying mechanisms of SUD development. However, many questions remain unresolved and future research should aim at expanding and refining the etiological model proposed in this overview. Hence, the development of improved standardized measures and the progressive embedding of psychoanalytic theories within empirical neuroscience might be rewarding goals for future research initiatives.

## Data Availability Statement

The original contributions presented in the study are included in the article/supplementary material; further inquiries can be directed to the corresponding author.

## Ethics Statement

This study was carried out in accordance with the recommendations of the ethics guidelines of the Medical University of Graz. The protocol was approved by the ethics committee of the Medical University of Graz. All subjects gave written informed consent in accordance with the Declaration of Helsinki.

## Author Contributions

JF and HU conceptualized the manuscript. JF wrote the first draft of the manuscript. HU proofread the manuscript. All authors contributed to the article and approved the submitted version.

## Conflict of Interest

The authors declare that the research was conducted in the absence of any commercial or financial relationships that could be construed as a potential conflict of interest.
